# A Logical Modeling of Severe Ignorance

**DOI:** 10.1007/s10992-022-09697-x

**Published:** 2023-04-11

**Authors:** S. Bonzio, V. Fano, P. Graziani, M. Pra Baldi

**Affiliations:** 1 Department of Mathematics and Computer Science, University of Cagliari, Cagliari, Italy; 2 Department of Pure and Applied Sciences, University of Urbino Carlo Bo, Urbino, Italy; 3 Artificial Intelligence Research Institute (IIIA), Spanish National Council of Research (CSIC), Bellaterra, Spain

**Keywords:** Ignorance, Three-valued modal logic, Bochvar external logic, Weak Kleene logic

## Abstract

In the logical context, ignorance is traditionally defined recurring to epistemic logic. In particular, ignorance is essentially interpreted as “lack of knowledge”. This received view has - as we point out - some problems, in particular we will highlight how it does not allow to express a type of content-theoretic ignorance, i.e. an ignorance of *φ* that stems from an unfamiliarity with its meaning. Contrarily to this trend, in this paper, we introduce and investigate a modal logic having a primitive epistemic operator **I**, modeling ignorance. Our modal logic is essentially constructed on the modal logics based on weak Kleene three-valued logic introduced by Segerberg (*Theoria, 33*(1):53–71, 1997). Such non-classical propositional basis allows to define a Kripke-style semantics with the following, very intuitive, interpretation: a formula *φ* is ignored by an agent if *φ* is neither true nor false in every world accessible to the agent. As a consequence of this choice, we obtain a type of content-theoretic notion of ignorance, which is essentially different from the traditional approach. We dub it *severe ignorance*. We axiomatize, prove completeness and decidability for the logic of reflexive (three-valued) Kripke frames, which we find the most suitable candidate for our novel proposal and, finally, compare our approach with the most traditional one.

## Data Availability

No data set has been used.

## Appendix

In this Appendix we provide the details of the proof of strong completeness for Bochvar (external) logic *B*_*e*_ (Theorem 7). Moreover, we also show that *B*_*e*_ is algebraizable. We start with a preliminary lemma.

### **Lemma 1**

The following elementary facts holds in *B*_*e*_:

1. $\vdash _{\mathsf {B}_{e}}J_{_0}(\varphi \lor \psi )\leftrightarrow J_{_0}\varphi \wedge _{_0}\psi $;
2. $\vdash _{\mathsf {B}_{e}}J_{_1}(\varphi \lor \psi )\leftrightarrow J_{_1}\varphi \vee J_{_1}\psi $;
3. $\vdash _{\mathsf {B}_{e}}J_{_{2}}(\varphi \lor \psi )\leftrightarrow (J_{_{2}}\varphi \wedge J_{_{2}}\psi )\vee (J_{_{2}}\varphi \wedge J_{_{2}}\neg \psi )\vee (J_{_{2}}\neg \varphi \wedge J_{_{2}}\psi ) $;
4. if *α* is an external formula, then $\vdash _{\mathsf {B}_{e}} \alpha \leftrightarrow J_{_2}\alpha $;
5. $\vdash _{\mathsf {B}_{e}}\neg J_{_2}\psi \leftrightarrow (J_{_0}\psi \vee J_{_1}\psi )$.

### *Proof*

Immediate by using Theorem 6. □

### **Lemma 2**

Let ${\Gamma }\cup \{\varphi \}\vdash _{\mathsf {B}_{e}}\psi $. Then ${\Gamma }\vdash _{\mathsf {B}_{e}}J_{_2}\varphi \to J_{_2}\psi $.

### *Proof*

By induction on the length of the derivation of *ψ* (from Γ). □

The class of *Bochvar algebras* is introduced by Finn and Grigolia [18, pp. 233-234] as algebraic semantics for *B*_*e*_.

### **Definition 3**

A Bochvar algebra $\mathbf {A}=\langle {A,\vee ,\wedge , \neg , J_{_0},J_{_1},J_{_2}, 0,1}\rangle $ is an algebra of type 〈2, 2, 1, 1, 1, 1, 0, 0〉 satisfying the following identities and quasi-identities:

1. *x* ∨ *x* ≈ *x*;
2. *x* ∨ *y* ≈ *y* ∨ *x*;
3. (*x* ∨ *y*) ∨ *z* ≈ *x* ∨ (*y* ∨ *z*);
4. (*x* ∧ (*y* ∨ *z*) ≈ ((*x* ∧ *y*) ∨ (*x* ∧ *z*));
5. ¬(¬*x*) ≈ *x*;
6. ¬1 ≈ 0;
7. ¬(*x* ∨ *y*) ≈¬*x* ∧¬*y*;
8. 0 ∨ *x* ≈ *x*;
9. $J_{_2}J_{_i} x\approx J_{_i} x$, for every *i* ∈{0, 1, 2};
10. $J_{_0}J_{_i} x\approx \neg J_{_i} x$, for every *i* ∈{0, 1, 2};
11. $J_{_{1}}J_{_i} x \approx 0$, for every *i* ∈{0, 1, 2};
12. $J_{_{i}}(\neg x)\approx J_{_{2}-i}x$, for every *i* ∈{0, 1, 2};
13. $J_{_{i}}x \approx \neg (J_{_{j}}x\vee J_{_{k}}x)$, for *i*≠*j*≠*k*≠*i*;
14. $J_{_{i}}x\vee \neg J_{_{i}}x \approx 1$, for every *i* ∈{0, 1, 2};
15. $((J_{_{i}}x\vee J_{_{k}}x)\wedge J_{_{i}}x)\approx J_{_{i}}x$, for *i*,*k* ∈{0, 1, 2};
16. $ x\lor J_{_{i}}x \approx x$, for *i* ∈{1, 2};
17. $J_{_{0}}(x\lor y)\approx J_{_{0}}x\wedge J_{_{0}}y$;
18. $J_{_{2}}(x\lor y)\approx (J_{_{2}}x\wedge J_{_{2}}y)\vee (J_{_{2}}x\wedge J_{_{2}}\neg y)\vee (J_{_{2}}\neg x\wedge J_{_{2}}y) $;
19. $J_{_0} x \approx J_{_0} y \& J_{_1} x \approx J_{_1} y \& J_{_2} x \approx J_{_2} y \Rightarrow x \approx y$.

We denote by ${\mathscr{B}}\mathcal {A}_{3}$ the class of Bochvar algebras. ${\mathscr{B}}\mathcal {A}_{3}$ forms a quasi-variety which is not a variety [18]. Recall that a class $\mathcal {K}$ of algebras is an algebraic semantics for a logic L provided that: Γ ⊩_*L*_*φ* iff $\{\tau (\gamma ): \gamma \in {\Gamma }\}\models _{Eq(\mathcal {K})} \tau (\varphi )$, where *τ* = {*φ*_*i*_(*p*) ≈ *ψ*_*i*_(*p*)} is a formula-equation transformer and $Eq(\mathcal {K})$ denotes the usual equational consequence relation relative to the class $\mathcal {K}$.

### **Theorem 4**

${\mathscr{B}}\mathcal {A}_{3}$ is an algebraic semantics for *B*_*e*_. In particular, ${\Gamma }\vdash _{\mathsf {B}_{e}}\varphi $ iff $\{\gamma \approx 1: \gamma \in {\Gamma }\}\models _{Eq({\mathscr{B}}\mathcal {A}_{3})} \varphi \approx 1$.

### *Proof*

(⇒) By induction on the length of the derivation of *φ* (from Γ), by checking that axioms (A1)-(A29) are evaluated to 1 in every Bochvar algebra **A** and that the rule (MP) preserves this property.

(⇐) We reason by contraposition. Suppose ${\Gamma }\nvdash _{\mathsf {B}_{e}}\varphi $ and provide a counterexample to such inference by constructing the Lindenbaum-Tarski algebra. Let Δ be the smallest set of formulas including Γ and closed under $\vdash _{\mathsf {B}_{e}}$ (from now on we will simply write ⊩ instead of $\vdash _{\mathsf {B}_{e}}$). For any pair of formulas, define

$$ \varphi\sim\psi \text{if and only if}\ \varphi\equiv\psi\in{\Delta}. $$

We claim that:

1. $\sim $ is a congruence on **F****m**;
2. $[1]_{\sim } = {\Delta }$;
3. The quotient algebra $\mathbf {Fm}_{/_{\sim }}$ is a Bochvar algebra.

1. It is easy to check that $\sim $ is an equivalence relation. To show that it is a congruence, we check the compatibility of $\sim $ with the operations in the type of ${\mathscr{L}}_{K^{e}}$.
  [¬] Suppose $\varphi \sim \psi $, then *φ* ≡ *ψ* ∈Δ, i.e. $\displaystyle \bigwedge _{i=0}^{2} J_{_{i}}\varphi \leftrightarrow J_{_{i}} \psi \in {\Delta }$, which is equivalent to $\displaystyle \bigwedge _{i=0}^{2} J_{_{2-i}}\varphi \leftrightarrow J_{_{2-i}} \psi \in {\Delta }$. Hence, in virtue of (A12), $\displaystyle \bigwedge _{i=0}^{2} J_{_i}(\neg \varphi )\leftrightarrow J_{_i}(\neg \psi )\in {\Delta }$, i.e. ¬*φ* ≡¬*ψ* ∈Δ, showing that $\neg \varphi \sim \neg \psi $.
  [$J_{_2}$] Suppose $\varphi \sim \psi $, thus *φ* ≡ *ψ* ∈Δ, i.e. $\displaystyle \bigwedge _{i=0}^{2} J_{_i}\varphi \leftrightarrow J_{_i}\psi \in {\Delta }$. In particular ${\Delta }\vdash J_{_2}\varphi \leftrightarrow J_{_2}\psi $. In virtue of (A9), we have $\vdash J_{_2}\varphi \leftrightarrow J_{_2}J_{_2}\varphi $ and $\vdash J_{_2}\psi \leftrightarrow J_{_2}J_{_2}\psi $, from which ${\Delta }\vdash J_{_2}J_{_2}\varphi \leftrightarrow J_{_2}J_{_2}\psi $, i.e. $J_{_2}J_{_2}\varphi \leftrightarrow J_{_2}J_{_2}\psi \in {\Delta } $ (as Δ is closed under consequences of ⊩). Analog reasoning, using (A10) and (A11), shows that $J_{_0}J_{_2}\varphi \leftrightarrow J_{_0}J_{_2}\psi \in {\Delta } $ and $J_{_1}J_{_2}\varphi \leftrightarrow J_{_1}J_{_2}\psi \in {\Delta } $, from which $J_{_2}\varphi \sim J_{_2}\psi $.
  [∨] Suppose $\varphi _{1}\sim \psi _{1}$ and $\varphi _{2}\sim \psi _{2}$. Then ${\Delta }\vdash J_{_0}\varphi _{1}\leftrightarrow J_{_0}\psi _{1}$ and ${\Delta }\vdash J_{_0}\varphi _{2}\leftrightarrow J_{_0}\psi _{2}$, hence ${\Delta }\vdash (J_{_0}\varphi _{1}\wedge J_{_0}\varphi _{2})\leftrightarrow (J_{_0}\psi _{1}\wedge J_{_0}\psi _{2})$. Applying Lemma 31-(1), we have ${\Delta }\vdash J_{_0}(\varphi _{1}\lor \varphi _{2})\leftrightarrow J_{_0}(\psi _{1}\lor \psi _{2})$, from which $J_{_0}(\varphi _{1}\lor \varphi _{2})\leftrightarrow J_{_0}(\psi _{1}\lor \psi _{2})\in {\Delta }$. Analog reasoning (using Lemma 31-(2,3)) allows to conclude $\varphi _{1}\lor \varphi _{2}\sim \psi _{1}\lor \psi _{2}$.
2. [$\subseteq $] Let $\psi \in [1]_{\sim }$. Then *ψ* ≡ 1 ∈Δ, i.e. $\displaystyle \bigwedge _{i=0}^{2} J_{_i} \psi \leftrightarrow J_{_i} 1\in {\Delta }$. In particular, $J_{_2}\psi \in {\Delta }$ (since $\vdash J_{_2} 1$) and $J_{_1}\psi \leftrightarrow 0\in {\Delta }$ (as $\vdash J_{_1} 1\leftrightarrow 0$), from which we deduce that *ψ* is an external formula, so, by Lemma 31-(4), *ψ* ∈Δ.
  [$\supseteq $] Let *ψ* ∈Δ. Observe that 1 ∈Δ (since ⊩ 1), hence, by Lemma 32, ${\Delta }\vdash J_{_2}\psi \leftrightarrow J_{_2} 1$, thus $J_{_2}\psi \leftrightarrow J_{_2} 1\in {\Delta }$. Moreover, ${\Delta }\vdash \neg J_{_2}\psi \leftrightarrow 0$ and, by Lemma 31, $\vdash \neg J_{_2}\psi \leftrightarrow (J_{_0}\psi \vee J_{_1}\psi )$, so ${\Delta }\vdash (J_{_0}\psi \vee J_{_1}\psi )\leftrightarrow 0$, from which ${\Delta }\vdash J_{_0}\psi \leftrightarrow 0 $ and $ {\Delta }\vdash J_{_1}\psi \leftrightarrow 0$; therefore ${\Delta }\vdash J_{_0}\psi \leftrightarrow J_{_0} 1 $ and $ {\Delta }\vdash J_{_1}\psi \leftrightarrow J_{_1} 1$ (as $\vdash J_{_0} 1\leftrightarrow 0$ and $\vdash J_{_1} 1\leftrightarrow 0$). This shows that *ψ* ≡ 1 ∈Δ, i.e. $\psi \in [1]_{\sim }$.
3. It is routine to check that $\mathbf {Fm}_{/_{\sim }}$ is indeed a Bochvar algebra.
  To provide a counterexample to the inference ${\Gamma }\nvdash _{\mathsf {B}_{e}}\varphi $, consider the Bochvar algebra $\mathbf {A}= \mathbf {Fm}_{/_{\sim }}$ and the homomorphism *h*: **F****m** →**A**, $h(\varphi )= [\varphi ]_{\sim }$. Since ${\Gamma }\subseteq {\Delta }$ and ${\Delta } = [1]_{\sim }$, then *h*(*γ*) = 1^**A**^, for each *γ* ∈Γ, but *h*(*φ*)≠ 1^**A**^ (since *φ*∉Δ).

□

Theorem 7 follows from Theorem 34 by observing that ${\mathscr{B}}\mathcal {A}_{3}$ is the quasi-variety generated by **W****K**^*e*^ ([18, Theorem 3.3]).

It is natural to wonder whether the quasi-variety of Bochvar algebras is simply *an* algebraic semantics for *B*_*e*_. Actually the relationship between *B*_*e*_ and the class ${\mathscr{B}}\mathcal {A}_{3}$ is tighter. Recall that a logic ⊩ is algebraizable with $\mathcal {K}$ as equivalent algebraic semantics (where $\mathcal {K} $ is a class of algebras of the same language as the logic ⊩) if there exists a map *τ* from formulas to sets of equations, and a map *ρ* from equations to sets of formulas such that the following conditions hold, for any pair of formulas *φ*,*ψ* and set of equations *E*.

- Γ ⊩ *φ* iff $\tau [{\Gamma }]\models _{Eq(\mathcal {K})}\tau (\varphi )$;
- $E\models _{Eq(\mathcal {K})} \varphi \approx \psi $ iff $\rho (E)\models _{Eq(\mathcal {K})}\rho (\varphi ,\psi )$;
- *φ* ⊣⊩ *ρ*(*τ*(*φ*));
- $\varphi \approx \psi =\joinrel \mathrel |\models _{Eq\left (\mathcal {K}\right )} \tau (\rho (\varphi ,\psi ))$.

Examples of algebraizable logics include, among many others, classical, intuitionistic logic, all substructural logics and global modal logics. Not all logics though are algebraizable: examples of non-algebraizable logics can be found in the realm of Kleene logics, such as the Logic of Paradox (see [37]), Paraconsistent weak Kleene (see [6]) and Bochvar internal logic (see [7–9]). The above definition of algebraizable logic can be drastically simplified: ⊩ is algebraizable with equivalent algebraic semantics $\mathcal {K}$ if and only if it satisfies either ALG1 and ALG4 (or, else ALG2 and ALG3).^1^

### **Theorem 5**

The logic *B*_*e*_ is algebraizable with ${\mathscr{B}}\mathcal {A}_{3}$ as equivalent algebraic semantics.

### *Proof*

Consider *τ* = {*φ* ≈ 1} and *ρ* = {*φ* ≡ *ψ*}. □

The usefulness of the above result will be explored in a fore-coming paper, focused on a deeper understanding of the properties of Bochvar algebras [10].
